# The adjuvant value of *Andrographis paniculata* in metastatic esophageal cancer treatment – from preclinical perspectives

**DOI:** 10.1038/s41598-017-00934-x

**Published:** 2017-04-12

**Authors:** Lin Li, Grace Gar-Lee Yue, Julia Kin-Ming Lee, Eric Chun-Wai Wong, Kwok-Pui Fung, Jun Yu, Clara Bik-San Lau, Philip Wai-Yan Chiu

**Affiliations:** 1 Department of Surgery, The Chinese University of Hong Kong, Shatin, New Territories Hong Kong; 2 Institute of Chinese Medicine and State Key Laboratory of Phytochemistry and Plant Resources in West China (CUHK), The Chinese University of Hong Kong, Shatin, New Territories Hong Kong; 3 School of Biomedical Sciences, The Chinese University of Hong Kong, Shatin, New Territories Hong Kong; 4 Department of Medicine and Therapeutics and State Key Laboratory of Digestive Disease, The Chinese University of Hong Kong, Shatin, New Territories Hong Kong

## Abstract

Esophageal cancer (EC) is the fourth and sixth leading cause of cancer-related deaths in China and United States, respectively. The dismal prognosis of EC is mainly attributed to distant metastases, which may not be overcome by chemotherapy alone. Hence, the use of alternative adjuvant treatments, such as herbal medicines, for metastatic EC remains a great desire of patients. Our previous study demonstrated the *in vivo* anti-tumor and *in vitro* anti-invasion activities of *Andrographis paniculata* (AP) in esophageal cancer. In the present study, the chemical constituents of absorbed AP components through human intestinal Caco-2 cell monolayer were verified for the first time. The anti-migratory activities and suppressive effects on metastasis-related factors such as HER2, MMP2, MMP9, TM4SF3, CXCR4 of the absorbed AP components were revealed in esophageal cancer cells EC-109. The anti-tumor and anti-metastatic effects of AP water extract (1600 mg/kg) were further confirmed in metastatic esophageal xenograft-bearing mice. Besides, AP water extract acted synergistically with cisplatin plus 5-fluorouracil on inhibiting tumor nodule growth (with combination index <0.7). Meanwhile, chemotherapeutics-induced side-effects could also be reduced by AP water extract. The present findings provide evidence on safety and advantages of the combined use of AP with chemotherapeutics in pre-clinical setting.

## Introduction

Esophageal cancer (EC) is the sixth leading cause of cancer-related death for male in United States^1^. Whilst being one of the four most common cancers diagnosed in China, the incidence is much higher than that in the United States^2^. This form of cancer is associated with a poor prognosis and the 5-year relative survival rate is only 10–15%^3^. Surgery currently offers the best treatment option for EC, however, postoperative recurrence rate is high due to cancer metastasis. Patients with EC are often accompanied by extensive metastasis to the celiac lymph nodes, liver, and lung^4^. Even though surgery followed by adjuvant chemotherapy and concurrent radiotherapy have shown some efficacies, the search for alternative treatments for metastatic EC is still desperately needed. Moreover, it is worth studying whether the side effects of chemotherapy and radiotherapy, including toxicity of organs, neutropenia, diarrhea, skin irritation, nausea, vomiting, intestinal discomfort etc.^5^, would be attenuated or eliminated by the use of complementary and alternative medicines.

Herbal medicines have been used for thousands of years, including some with anticancer activity and low toxicity. Modern pharmacological research has increasingly provided evidences for herbal medicines to be potential alternative therapeutics for the treatment of various cancers (in both clinical and pre-clinical studies)^6, 7^. Recently, consumption of herbal remedies either alone or in combination with conventional chemotherapeutic agents has become a popular practice in patients suffering from cancer^8^. Therefore, the evaluation of the therapeutic efficacy and/or toxicity is necessary.

Oral administration of herbal drugs is the most convenient route. However, oral drug absorption is complex due to the inherent characteristics, transport and metabolism of drugs, etc. Hence, transmembrane absorption is the first consideration in efficient systemic availability of drugs administered through the gastrointestinal tract. Since Caco-2 cells express a wide range of enzymes and transporters which are similar to those of intestinal endothelium cells, the application of the Caco-2 monolayer model to mimic the microvilli and enterocytes of the human small intestine, has become a well known *in vitro* model^8, 9^. The utilization of Caco-2 transport model, which was recommended as a pre-clinical integral component of the Biopharmaceutics Classification System by FDA, can be used to investigate the gastrointestinal absorption, permeability and drug-drug interactions^8^. Recently, Caco-2 transport model was also widely used to study the absorption and permeability of herbal medicines which contain numerous phytochemical compounds^10–12^.

The herbal medicine *Andrographis paniculata* (AP) Wall (family Acanthaceae) is one of the most popular plants used traditionally for treatment of diseases such as cancer, influenza, diabetes, hypertension, ulcer, etc. in the continents of Asia, America and Africa^13^. Modern pharmacological studies suggested that AP had the anti-tumor and immunomodulatory effects *in vitro* and *in vivo* ^14^. The secondary metabolites including diterpenes, lactones and flavonoids existing in AP were responsible for its activities^13, 15^.

Our previous studies revealed that AP possessed potential anti-tumor and anti-metastatic effects on EC^16^. Given that the Caco-2 model is considered the most common *in vitro* model for studying and predicting intestinal drug absorption characteristics, it was employed for the investigation of absorption of the water extract of AP (APW) in our present study. Seven chemical constituents, including five diterpenes (andrographolide, neoandrographolide, deoxyandrographolide, andropanoside and dehydroandrographolide), as well as two flavonoids (5-hydroxy-7,8-dimethoxyflavone and 5-hydroxy-7,8-dimethoxyflavanone) were detected and quantified in components transported through the Caco-2 monolayer. Meanwhile, the components transported through the Caco-2 monolayer (absorbed AP components, AAPC), which mimics components absorbed by the human intestine, were collected for evaluation of its anti-metastatic effects on human esophageal cancer cells EC-109. In addition, the regulating effects of AAPC on some metastasis-related genes and proteins in EC-109 cells were also studied. Finally, the combined efficacy of AP and standard chemotherapeutics cisplatin and 5-fluorouracil (5-FU)^17^ was explored in the intraperitoneal esophageal tumor xenograft-bearing mice.

## Results

### Identification and quantification of chemical constituents in AAPC

**Figure 1:**
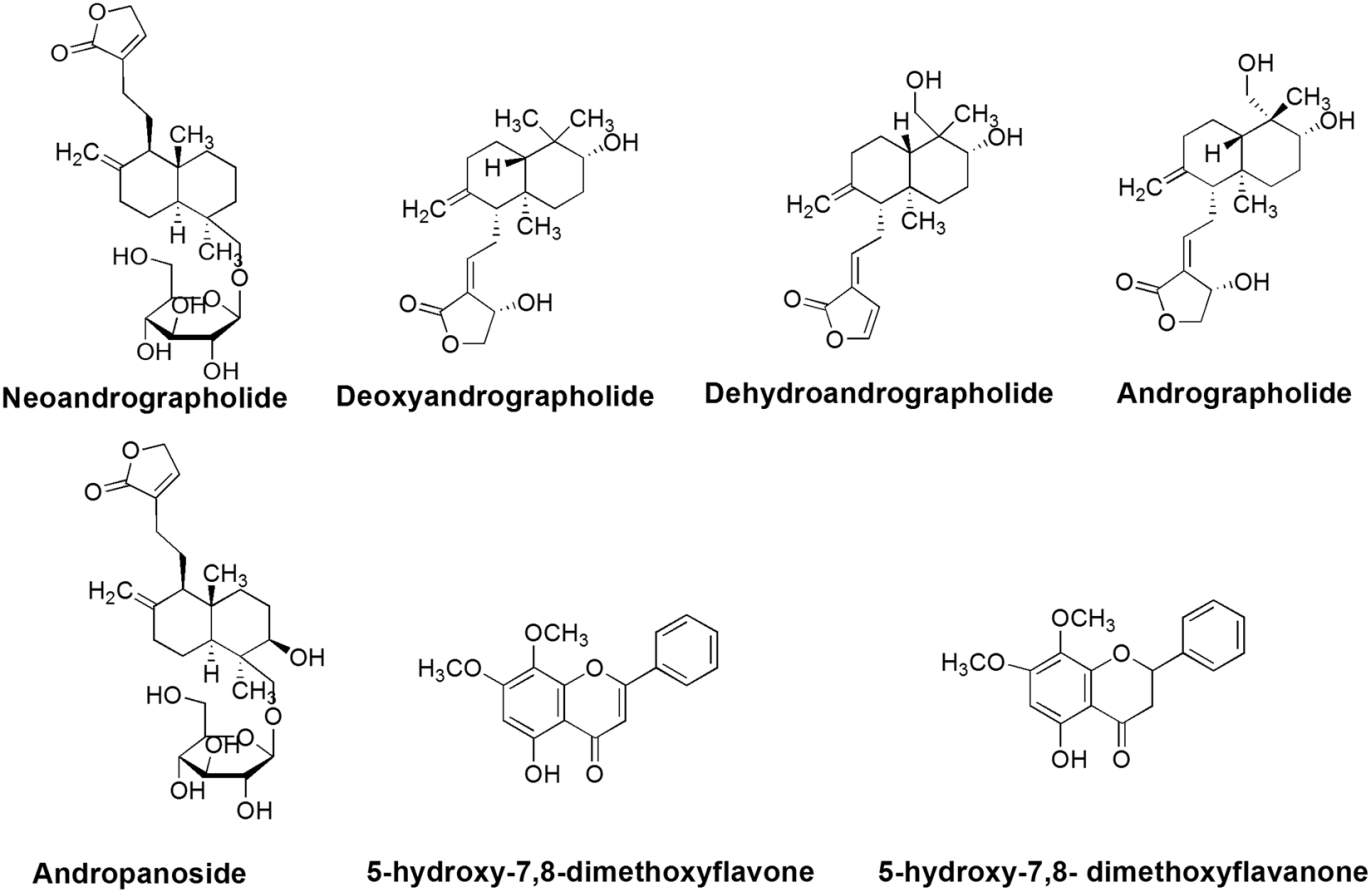
The structures of seven chemical constituents identified and quantified in absorbed AP components.

*In vitro* absorption study showed that seven known chemical constituents were identified in the basolateral side of the monolayer after addition of 400–6400 μg/ml of APW on the apical side of Caco-2 cell monolayer, namely, andrographolide, andropanoside, dehydroandrographolide, deoxyandrographolide, neoandrographolide, 5-hydroxy-7,8-dimethoxyflavone and 5-hydroxy-7,8-dimethoxyflavanone (Fig. 1). These were considered as AAPC, and their contents were quantified by HPLC or LC-MS (Table 1).

**Table 1:** The content of seven chemical constituents found in absorbed AP components from 400–6400 μg/ml APW.

APW concentration (μg/ml)	400	800	1600	3200	6400
Chemical constituents (μg/ml)					
Andrographolide	13.81 ± 1.586	27.41 ± 4.684	57.67 ± 2.351	97.16 ± 18.8	131.9 ± 33.47
Dehydroandrographolide	7.092 ± 1.131	14.07 ± 2.569	27.66 ± 2.595	50.36 ± 7.702	84.91 ± 12.82
Deoxyandrographolide	2.646 ± 0.4549	5.234 ± 1.278	10.17 ± 1.989	16.82 ± 3.736	25.42 ± 5.342
Andropanoside	1.871 ± 0.3119	3.925 ± 0.7175	8.266 ± 0.9118	15.48 ± 2.598	30.21 ± 3.570
Neoandrographolide	0.3192 ± 0.5529	0.6022 ± 1.043	2.061 ± 1.958	4.271 ± 3.112	13.15 ± 7.774
5-hydroxy-7,8-dimethoxyflavone	0	0	0.3948 ± 0.0363	0.6884 ± 0.0658	1.293 ± 0.1752
5-hydroxy-7,8-dimethoxyflavanone	0.4073 ± 0.0927	0.8625 ± 0.193	1.794 ± 0.1534	3.392 ± 0.2757	6.607 ± 0.7153

Chemical constituents of APW detected and quantified in the buffer from the basolateral side of intestinal Caco-2 cell monolayer. Data are expressed as the mean ± S.D. of three independent experiments.

### Cytotoxic effects of AAPC in EC-109 cells

**Figure 2:**
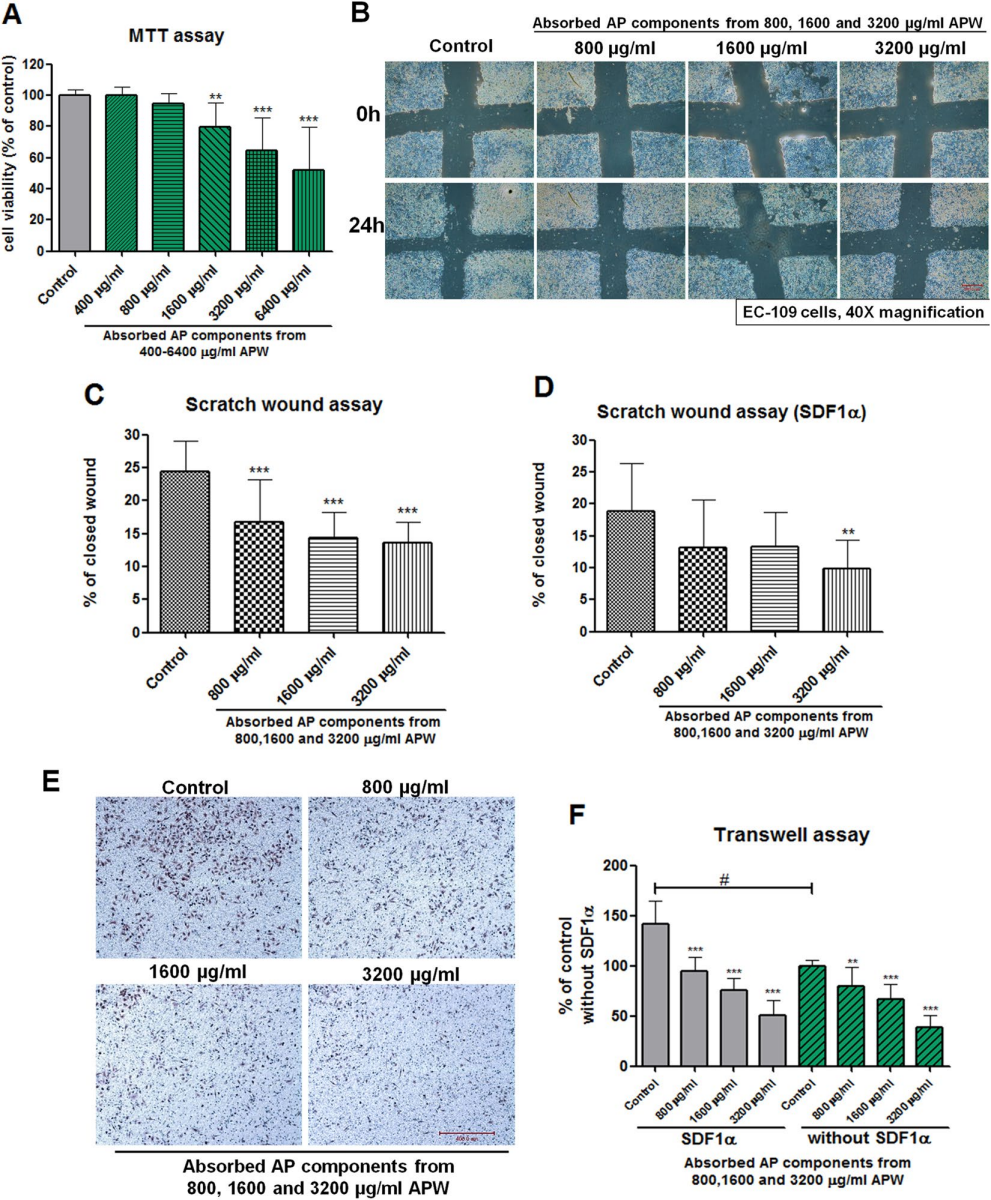
The cytotoxicity and anti-migratory effect of absorbed AP components in EC-109 cells. (**A**) Results showed that when 1600–6400 μg/mL of APW were added on the apical side of Caco-2 cell monolayer, the absorbed AP components collected on the basolateral side of the monolayer could significantly inhibit the growth of EC-109 cells after 48 h incubation (n = 3–4, mean + SD). (**B**–**D**) The anti-migration effect of absorbed AP components (AAPC) by scratch wound assay. (**B**) The representative photomicrographs showing human esophageal squamous carcinoma cells EC-109 migrated across the scratch wound in the presence or absence of AAPC for 24 h treatment. (**C** and **D**) The results are expressed as the percentage of closed wound area (mean + SD of three independent experiments with two wells each). (**E** and **F**) Effects of absorbed AP components (AAPC) on migration of EC-109 in Boyden chambers. (**E**) Representative photomicrographs showing the stained cells on the lower side of the membrane. The cells in the upper chambers were treated with different doses of AAPC. After 24 h incubation, those cells migrated to the lower chambers were stained and the numbers were counted. (**F**) Quantification of migration of EC-109 cells. Results are expressed as the mean percentage of control (mean + S.D. of three independent experiments with two wells each). Differences between AAPC treated groups and vehicle control group were determined by one-way ANOVA followed by *post-hoc* Dunnett’s test. **p < 0.01, ***p < 0.001 comparing AAPC treated groups with vehicle control. Difference between vehicle control groups with and without SDF1α was determined by Student’s t-test, ^#^ *p* < 0.001.

The AAPC (collected on the basolateral side of the Caco-2 cell monolayer after addition of 1600–6400 μg/ml APW on the apical side) significantly inhibited the growth of EC-109 cells after 48 h incubation (Fig. 2A). The inhibitory effects showed a concentration dependent manner.

### Inhibitory effects of AAPC on migration ability of EC-109 cells

Cancer cell migration and invasion are essential processes in cancer metastasis. Apart from anti-proliferative effects, the role of AAPC (at non-cytotoxic dose ranges) on migration of cancer cells was also explored. In the scratch wound assay, the closed wound areas in wells treated with AAPC from 800, 1600 and 3200 μg/ml APW were significantly smaller than those of control wells after incubation for 24 h (*p* < 0.001). The cell motility was decreased in a concentration-dependent manner (Fig. 2B and C). Moreover, AAPC also demonstrated inhibitory effects on SDF1α mediated migration in EC-109 cells (*p* < 0.01) (Fig. 2D).

In addition, the effects of AAPC treatment on cell migration were evaluated using a modified Boyden chamber assay (Fig. 2E). In control wells, lots of cancer cells migrated from the upper to the lower chamber through the membrane after 24 h incubation, with the lower chamber containing culture medium supplemented with 10% v/v FBS as chemoattractant. As shown in Fig. 2E and F, the cell migration significantly increased by 41.9% after addition of SDF1α (*p* < 0.001). In the presence of AAPC from 800, 1600 and 3200 μg/ml APW, cell invasion of EC-109 cells reduced by 20.1%, 33.6% and 61.0%, respectively. Meanwhile, after exposure to SDF1α, cell invasion reduced by 33.4%, 46.6% and 64.6%, respectively, in the presence of AAPC from 800, 1600, and 3200 μg/ml APW. Hence, AAPC was shown to be able to inhibit SDF1α-mediated cell migration in EC-109 cells and the cells exposed to SDF1α were more sensitive to the inhibitory effect of AAPC than those without SDF1α stimulation.

### Suppressive effect of AAPC on phosphorylated-NFκB expression

**Figure 3:**
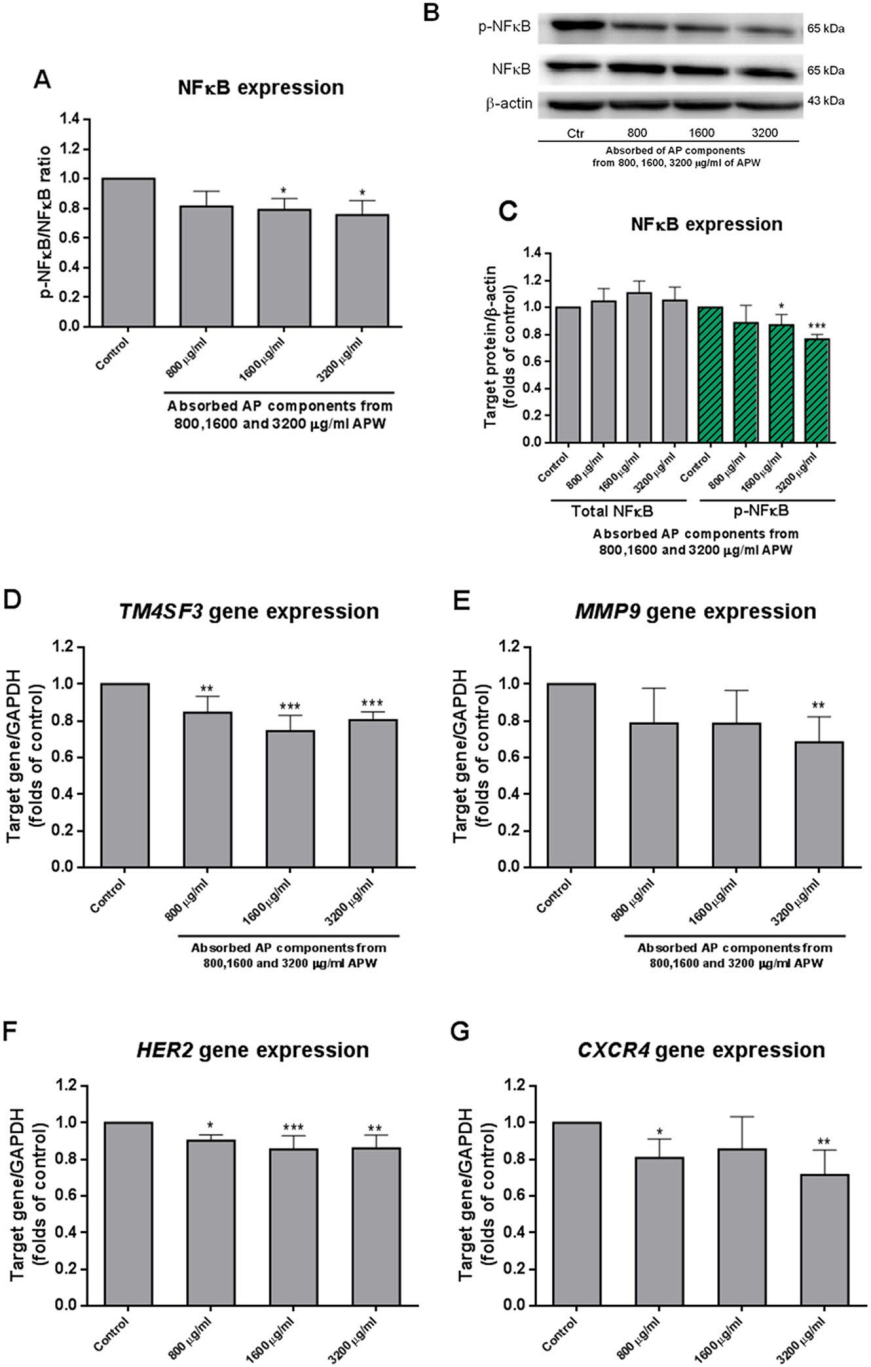
Effect of absorbed AP components from 800, 1600 and 3200 μg/ml APW on NFκB expression and the expression of metastasis-related genes. (**A**) NFκB ELISA assay. The OD values were normalised with the corresponding protein concentrations. Each column represents the mean + S.D. in duplicates of three independent experiments. (**B**) Western blot analysis of p-NFκB and NFκB expression. Representative immunoblots showing the effects of absorbed AP components from 800, 1600 and 3200 μg/ml APW on EC-109 cellular expression of p-NFκB and NFκB. Actin expression was determined to confirm equal protein loading. (**C**) The histogram showed quantified results of protein levels, which were adjusted with corresponding β-actin protein level and expressed as folds of control (mean fold of control + S.D. of three independent experiments). Differences between the treatment groups and the control group were determined by one way ANOVA followed by *post-hoc* Dunnett’s test, *p < 0.05, ***p < 0.001 as compared with vehicle control. (**D**–**G**) Real-time PCR showed the expression of metastasis-related genes. EC-109 cells were treated with absorbed AP components from 800, 1600 and 3200 μg/ml APW for 24 h and then the cells were collected for RNA extraction. Quantitative real time PCR analyses of mRNA of *TM4SF3*, *MMP9*, *HER2* and *CXCR4* genes were shown. Data were normalized to corresponding *GAPDH* expressions as internal control. The results of mRNA expressions are expressed as fold of control (mean fold of control + S.D. from 3 independent experiments). Differences among all groups were determined by one-way ANOVA followed by *post-hoc* Tukey’s multiple comparison test, *p < 0.05, **p < 0.01, ***p < 0.001 as compared with vehicle control.

The data from ELISA assay demonstrated the ratio of p-NFκB/NFκB significantly decreased after AAPC treatment (*p* < 0.05 for AAPC from 1600 or 3200 μg/ml APW) (Fig. 3A). Our results from western blotting also showed that AAPC significantly decreased the expression of p-NFκB (pS536) compared to control group after 24 h treatment (*p* < 0.05 for AAPC from 1600 μg/ml APW, *p* < 0.001 for AAPC from 3200 μg/ml APW). Meanwhile, no significant change of the expression of total NFκB was observed after treatment with AAPC for 24 h (Fig. 3B and C).

### Regulatory effects of AAPC on the mRNA and protein expression of metastasis-related factors in EC-109 cells

**Figure 4:**
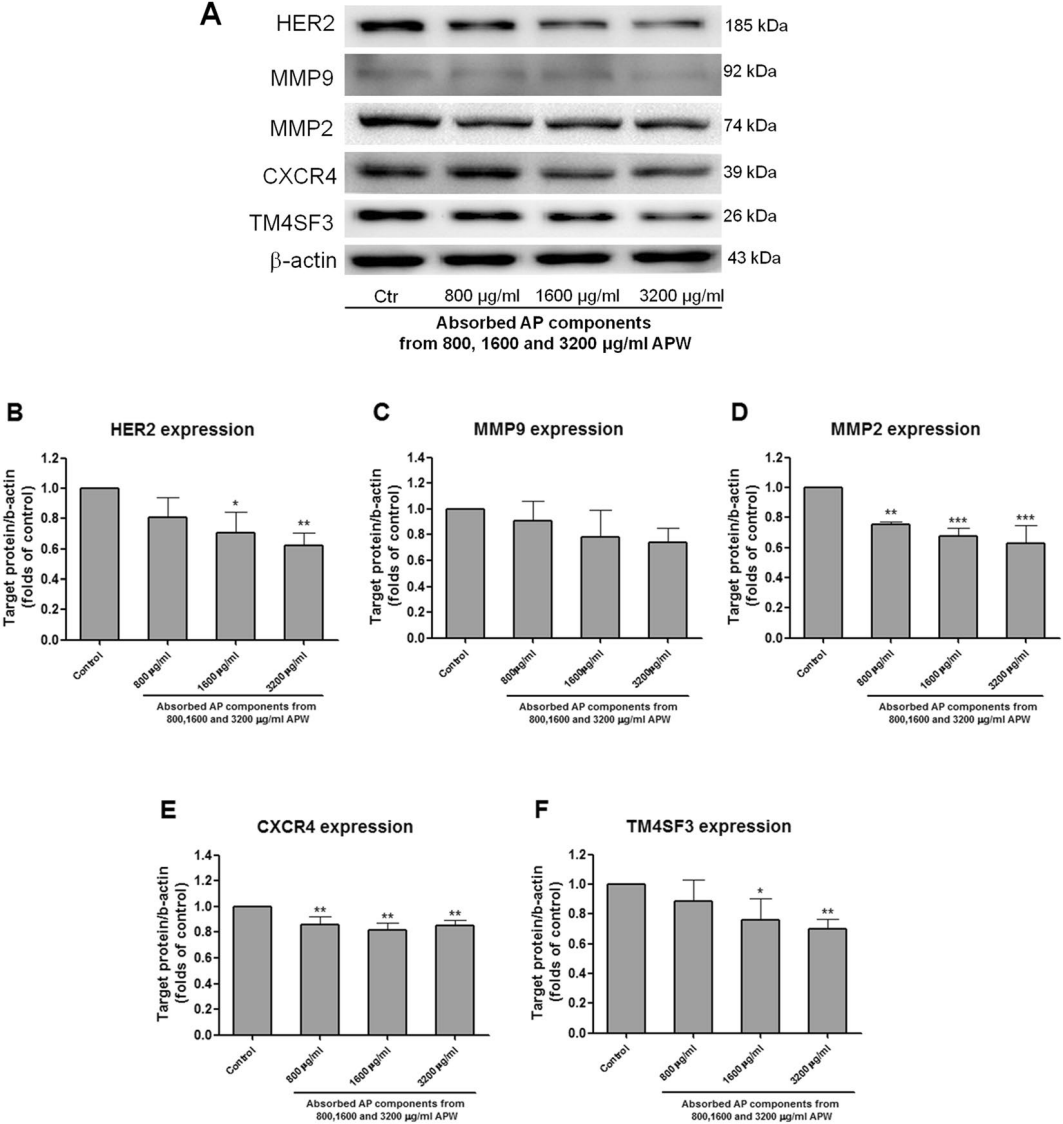
Western blot analysis of the expression of metastasis-related proteins. (**A**) Representative immunoblots showing the effects of absorbed AP components from 800, 1600 and 3200 μg/ml APW on EC-109 cellular expression of HER2, MMP9, MMP2, CXCR4, TM4SF3. Actin expression was determined to confirm equal protein loading. (**B**–**F**) The histograms showed quantified results of protein levels, which were adjusted with corresponding β-actin protein level and expressed as folds of control (mean fold of control + S.D. of three independent experiments). Differences among all groups were determined by one-way ANOVA followed by *post-hoc* Tukey’s multiple comparison test, *p < 0.05, **p < 0.01, ***p < 0.001 as compared with vehicle control.

The effects of AAPC on the expression of metastasis-related genes *TM4SF3, MMP9*, *CXCR4* and *HER2* in EC-109 cells were examined using real-time PCR. Results showed that the mRNA expressions of *TM4SF3* and *MMP9* were significantly decreased in a concentration-dependent manner after AAPC exposure (Fig. 3D and E). Meanwhile, the expressions of *HER2* and *CXCR4* were also significantly inhibited after AAPC treatment (Fig. 3F and G). The results of western blot showed that the protein expressions of HER2, MMP2, MMP9, CXCR4 and TM4SF3 were dose-dependently suppressed by AAPC after 24 h treatment (Fig. 4A). The quantifications of the decreases were shown in Fig. 4B–F. The suppressive effects on HER2, TM4SF3 (*p* < 0.05 for AAPC from 1600 μg/ml APW, *p* < 0.01 for AAPC from 3200 μg/ml APW), MMP2 (*p* < 0.01 for AAPC from 800 μg/ml APW, *p* < 0.001 for AAPC from 1600 or 3200 μg/ml APW) and CXCR4 (*p* < 0.01 for AAPC from 800, 1600 or 3200 μg/ml APW) were statistically significant when compared with control. While the suppressive effect on MMP9 protein expression was relatively mild which was not significantly differ from control (*p* = 0.2 for AAPC from 3200 μg/ml APW).

### Improved survival of intraperitoneal esophageal tumor xenograft-bearing mice after treatment with APW and/or chemotherapeutics

**Figure 5:**
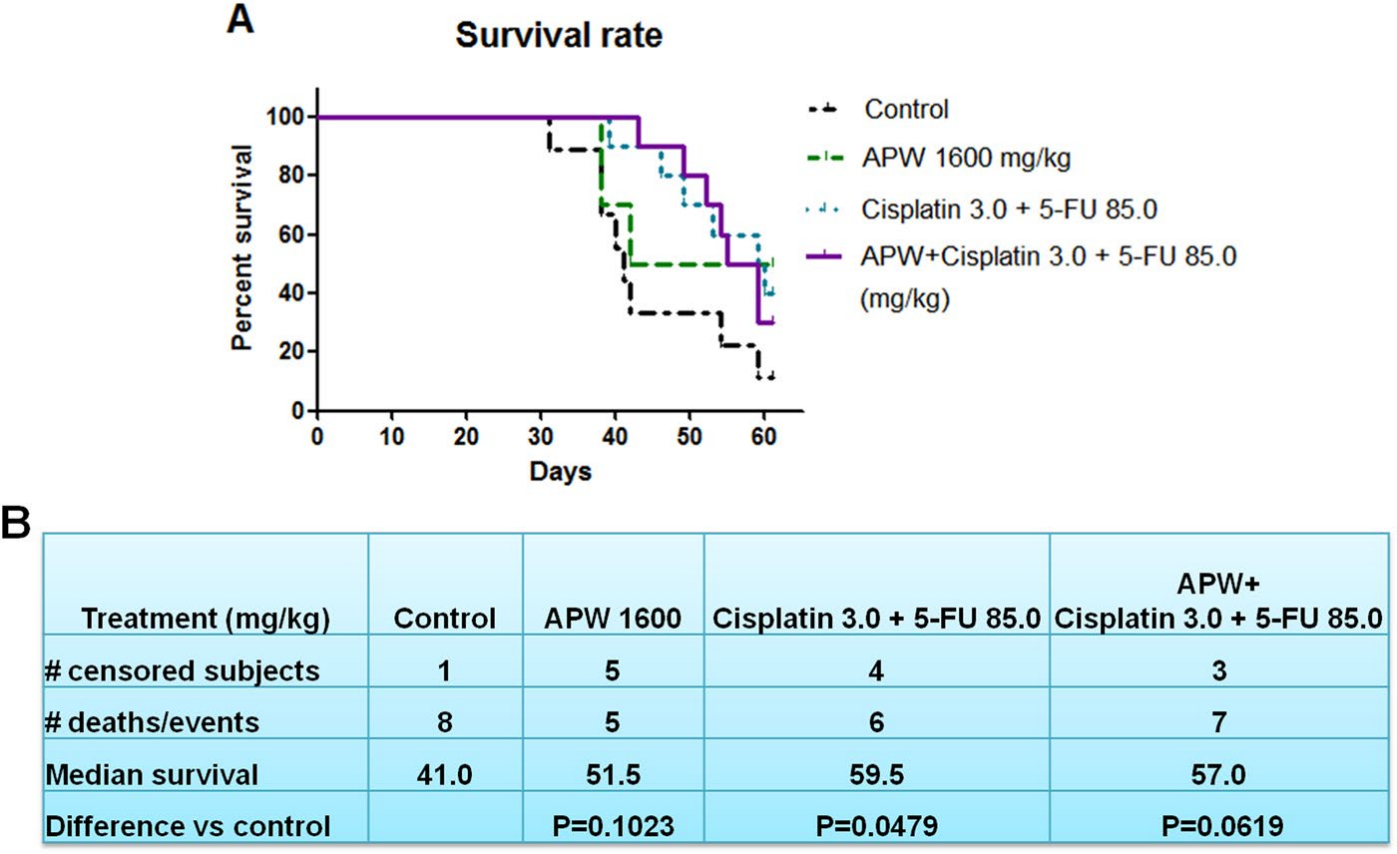
Treatments with APW alone, the combination of cisplatin and 5-FU and the combination of APW, cisplatin and 5-FU resulted in prolonged survival in mice inoculated with EC-109 cells intraperitoneally. Differences among treatment groups were compared using the LogRank test (n = 9–10).

Since AAPC showed promising results on *in vitro* studies, further animal studies were carried out. The intraperitoneal esophageal tumor xenograft model has been successfully established. The results showed that survival was prolonged by APW, Cisplatin + 5-FU and APW + Cisplatin + 5-FU as compared to control treatment in nude mice with EC-109 xenografts (Fig. 5A). The median survivals (95% confidence) were 41.0 days for control group, 51.5 days for APW group, 59.5 days for Cisplatin + 5-FU group, and 57.0 days for APW + Cisplatin + 5-FU group. The survivals of the Cisplatin + 5-FU and APW + Cisplatin + 5-FU groups were longer than control group (Fig. 5B).

### APW treatment enhanced the inhibitory effects of chemotherapeutics on tumor growth

**Figure 6:**
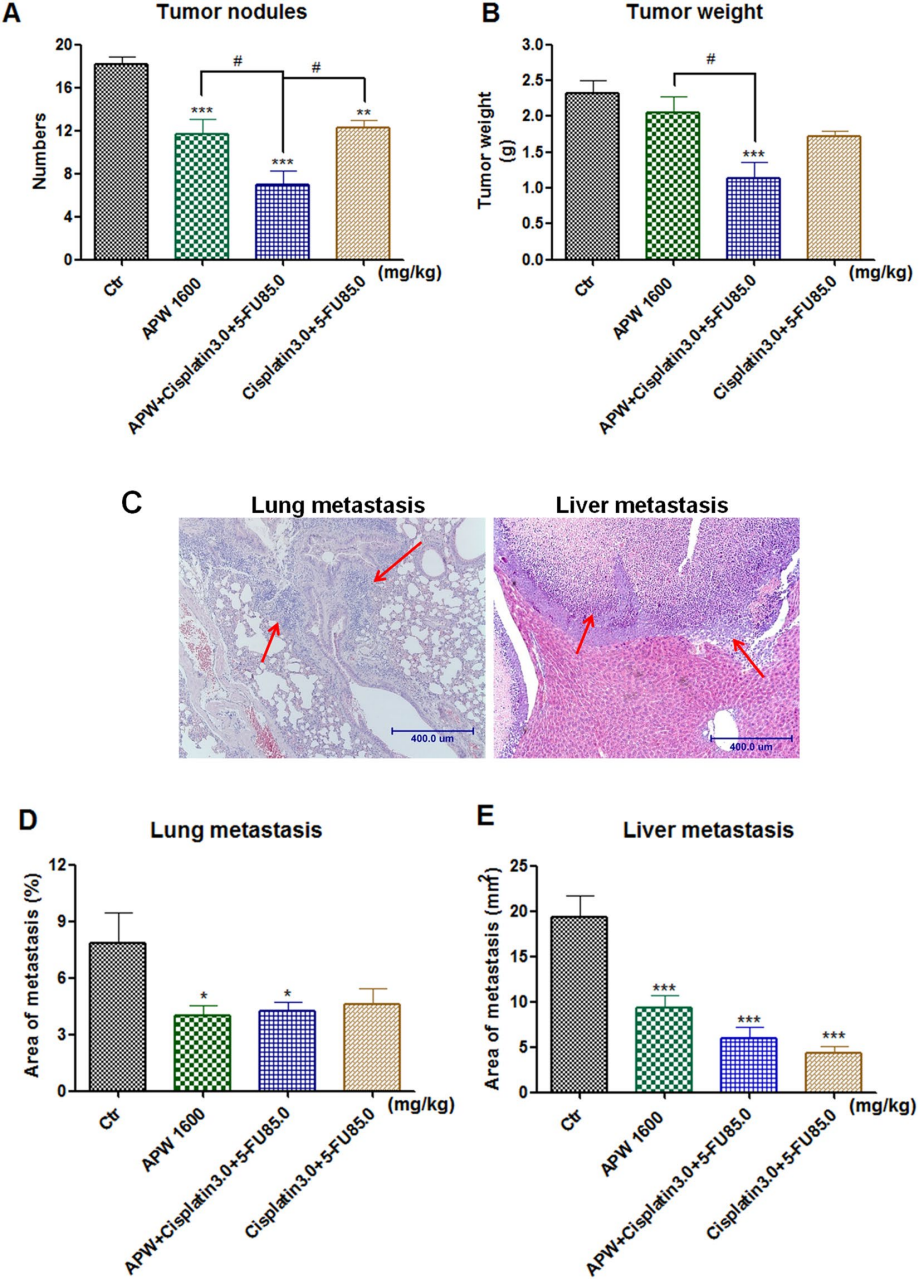
(**A** and **B**) The anti-tumor efficacies and (**C**–**E**) anti-metastatic effects of APW alone, the combination of cisplatin and 5-FU and the combination of APW, cisplatin and 5-FU in EC-109 intraperitoneal xenograft-bearing nude mice. (**A**) The numbers of tumor nodules (diameter greater than 2 mm) were counted. (**B**) All tumors collected from intraperitoneal cavity of each mouse were weight. (**C**) The representative photos of micro-metastasis in lungs and large nodules in liver with arrows showing the tumor cells. (**D**) The histograms represented the tumor burden in lungs as assessed by histological analysis, and expressed as an average percentage each group. (**E**) The histograms showed the tumor burden in liver, and expressed as an average tumor area per group in absolute units (mm^2^). Data are expressed as the mean + S.E.M. (n = 10–13), *p < 0.05, **p < 0.01, ***p < 0.001 as compared with control; ^#^ *p* < 0.05 as compared among treatment groups by one-way ANOVA followed by *post-hoc* Tukey’s multiple comparison test.

Since administration of APW showed potential benefits on the survival of intraperitoneal esophageal tumor xenograft-bearing mice, further experiments to explore the underlying principles were carried out. In the third week after inoculating cells into peritoneal cavity, malignant ascites, tumor nodules on the omenta and mesentery were observed. *In vivo* study showed that the number of tumor nodules was significantly decreased in tumor-bearing mice treated with APW for 21 days (Fig. 6A, *p* < 0.001). Besides, APW treatment further enhanced the inhibitory effects of Cisplatin + 5-FU on the number of tumor nodules (Fig. 6A, *p* < 0.05). In addition, tumor weights were significantly decreased in the group treated with APW + Cisplatin + 5-FU by 51.2%, when compared with control group (mean tumor weight decreased from 2.313 to 1.128 g). Meanwhile, tumor weights in the group treated with Cisplatin + 5-FU decreased by 25.9% only compared with control group (mean tumor weight decreased from 2.313 to 1.714 g) (Fig. 6B). The combination index values of APW with chemotherapeutics in tumor nodule number and tumor weight were 0.68 and 0.57, respectively. Results suggested synergism between APW and chemotherapeutics in terms of suppression of tumor growth and metastasis.

### APW treatment inhibited liver and lung metastases in intraperitoneal esophageal xenograft-bearing mice

Apart from tumor nodules and weights, tumor burden of the livers and lungs were also assessed. Micro-metastasis in lung and liver as well as large nodules in liver were observed (micro-metastasis in lung and large nodules in liver shown in red arrows, Fig. 6C). Tumor burden in the lungs were found to decrease by 49.4% (lung tumor burden decreased from 7.85% to 3.97%) in the APW-treated group when compared with control group, indicating that APW was effective in inhibiting metastasis to the lungs (Fig. 6D). At the same time, APW treatment showed a comparable reduction of lung metastasis with Cisplatin + 5-FU treatment, which was employed as standard chemotherapeutics clinically. In addition, the tumor burden in the liver was also evaluated. Data from H&E staining showed that the tumor burden in the livers was attenuated in all of the treated groups comparing with the control group (Fig. 6E). APW treatment reduced the tumor burden of the livers by 52.0% vs control group (liver tumor burden decreased from 19.39 to 9.31 mm^2^). However, APW + Cisplatin + 5-FU did not show further inhibitory effects on the tumor burden of lungs and livers, comparing with either APW alone or Cisplatin + 5-FU treatment groups (Fig. 6D and E).

### APW treatment improved the side-effects of chemotherapeutics

**Figure 7:**
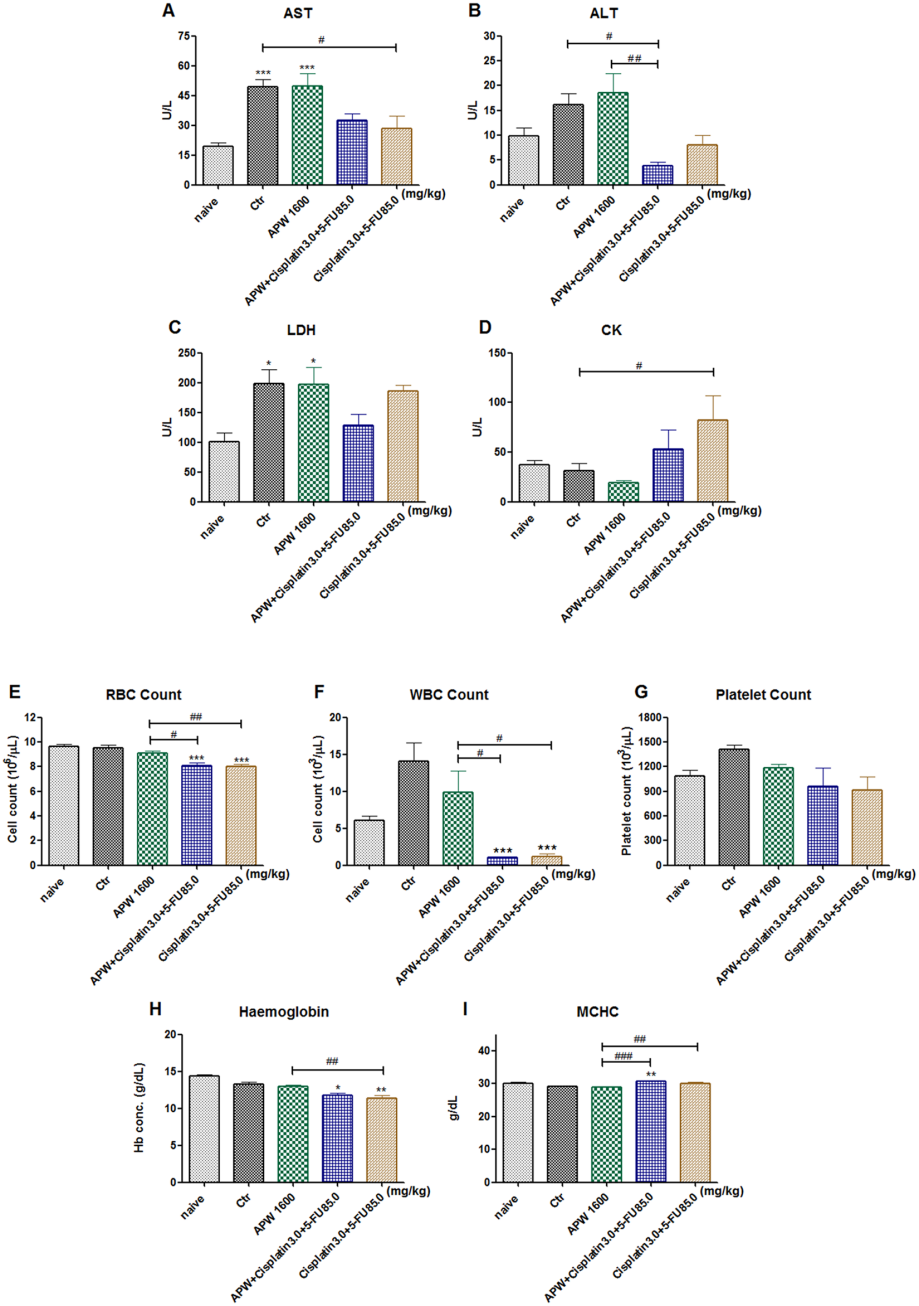
The treatment effects on plasma enzymes, blood cells count and haematological parameters of mice. (**A**–**D**) Effects of APW alone, the combination of cisplatin and 5-FU and the combination of APW, cisplatin and 5-FU treatments on the levels of plasma enzymes AST, ALT, LDH and CK in EC-109 intraperitoneal xenograft-bearing nude mice after 20 days of treatment. Data are expressed as the mean + S.E.M. of 10–13 samples. ^*^ *p* < 0.05, ^***^ *p* < 0.001 as compared with naïve; ^#^ *p* < 0.05 as compared with control by one-way ANOVA followed by *post-hoc* Tukey’s multiple comparison test. (**E**–**I**) Effects of APW alone, the combination of cisplatin and 5-FU and the combination of APW, cisplatin and 5-FU treatments on blood cells count and haematological parameters of mice. Results were expressed as mean + S.E.M. of 5 mice each group. *p < 0.05, **p < 0.01, ***p < 0.001 when treatment groups compared with control group. ^#^ *p* < 0.05, ^##^ *p* < 0.01, ^###^ *p* < 0.001 as compared among treatment groups by one-way ANOVA followed by *post-hoc* Tukey’s multiple comparison test.

To further estimate whether APW treatments damage the functions of the heart and liver, levels of muscle- and liver-specific enzymes in plasma were assessed. Results showed that the activities of liver specific plasma enzymes AST and LDH in the control group were significantly higher than those in naive group (AST, *p* < 0.001; LDH, *p* < 0.05; Fig. 7A,B and C), demonstrating that tumor burden of livers indeed caused liver damage in nude mice. ALT level in APW + Cisplatin + 5-FU treated group was significantly decreased comparing with either control group or APW alone group (Fig. 7B). Although there was no significant change on the activities of AST and LDH after 21 days of APW treatment, the enzyme levels were reduced in APW + Cisplatin + 5-FU treated mice as compared with control group. The results demonstrated that APW + Cisplatin + 5-FU treatment did not increase the burden of the livers, but even improved the functions of them. The activity of muscle-related enzyme CK was significantly higher in Cisplatin + 5-FU treated mice comparing with control group (*p* < 0.05). Meanwhile, no significant difference in the level of CK was found among the control, APW and APW + Cisplatin + 5-FU groups, suggesting that APW treatment not only has no obvious toxicity to the heart, but also attenuated the toxicities of the heart induced by chemotherapeutics (Fig. 7D).

Complete blood count was also employed for evaluation of the side effects of treatment. Our results showed that Cisplatin + 5-FU treatment significantly decreased the count of RBC (*p* < 0.001) and WBC (*p* < 0.001), as well as haemogloblin concentration (*p* < 0.01). The reduction of platelet count was also observed in Cisplatin + 5-FU group. In contrast, APW treatment did not affect these parameters (Fig. 7E–H). Nevertheless, pancytopenia and anemia were not improved after treatment of APW combined with Cisplatin + 5-FU, except for a slight increase in the level of MCHC (Fig. 7I).

## Discussion

Previous studies reported that the main active component of APW was andrographolide, whose bioavailability was poor^18^. Therefore, the Caco-2 transport model was employed to mimic the gastrointestinal absorption and permeability^8, 9^. For the first time, our present study showed that five diterpenes and two flavonoids (Fig. 1 and Table 1) could transport through the Caco-2 monolayer. Furthermore, we also found absorbed components of AP (AAPC) were capable to exert anti-invasion and anti-motility on esophageal cancer cells as shown in Fig. 2. Therefore, other components in addition to andrographolide might be responsible for the anti-metastatic effects. Due to the limitation of commercially available chemical markers, there were only seven compounds identified and quantified in AAPC in the present study. Although the major chemical constituents of APW had already been included, we still believed that more active components from APW were absorbed.

Esophageal cancer (EC) is known as a highly aggressive neoplasm mainly due to its strong invasive and metastatic behavior^4^. Previous study demonstrated that the over-expression of TM4SF3, a cell surface glycoprotein, in EC conferred advantage to cancer cell motility^19^. Our present results showed that the suppression of TM4SF3 expression by AAPC (Figs 3D and 4F) was possibly responsible for its inhibitory activities on the motility of EC cells. In addition, the chemokine SDF1α receptor, namely CXCR4, was also involved in the regulation of invasion, angiogenesis and metastasis in many types of tumors including EC^20^. CXCR4-positive cells were inclined to migrate to lymph nodes^21^ and showed significant positive association with MMP9 expression in EC^22^. SDF1α, the ligand of CXCR4, plays important role in tumor growth, angiogenesis and metastasis of different types of cancers through a CXCR4 dependent mechanism^21, 23^. Our data demonstrated that the addition of SDF1α stimulated the migration of EC cells and AAPC could inhibit such induced migration. In addition, the inhibitory effect of AAPC was stronger in the cells exposed to SDF1α than that in the cells without SDF1α exposure (Fig. 2). The results of western blotting and real-time PCR showed that AAPC inhibited CXCR4 and MMP9 expression (Figs 3 and 4). Among the human epithelial growth factor receptors, HER2 is the over-expressive oncogene and plays an important role in tumor growth and progression^24^. Previous study by Gros *et al*. found that HER2-mediated invasion was dependent on the up-regulation of CXCR4 in EC. The combined inhibition of HER2 and CXCR4 by trastuzumab and AMD3100 led to further reduction for primary tumor growth^24^. Therefore, the simultaneous inhibition of HER2 and CXCR4 by AAPC treatment enhanced its anti-tumor and anti-metastatic effects. Among the previously reported metalloproteinases (MMPs), MMP9 and MMP2 were key enzymes that contributed to the degradation of extracellular matrix components and type IV collagen. The MMP9 promoter region contains one NFκB binding site, which means the activation of MMP9 in cancer progression may partly be derived from NFκB transcription factor^25–27^. Our results showed that the phosphorylation of NFκB and MMP9 expression were inhibited by AAPC as shown in Figs 3 and 4. Therefore, one potential pathway whereby AAPC inhibited migration and invasion of EC-109 cells was regulated through the NFκB-mediated regulation of MMP9 expression. Cancer metastatic pathways are complex and still poorly understood^28, 29^. Our present study demonstrated for the first time that the suppression of TM4SF3, HER2, CXCR4, MMP2 and the NFκB-mediated regulation of MMP9 expression were involved in the anti-invasion and anti-migration effects of AAPC on EC-109. However, the correlation among the expression of these metastasis-related genes and proteins has not been elaborated and needs further investigation.

In view of AAPC showing very effective activities on anti-invasion and anti-migration *in vitro*, we further evaluated the anti-metastatic effects in intraperitoneal esophageal tumor xenograft-bearing mice. In survival experiments, on the 30^th^ day after esophageal squamous carcinoma cells inoculation, mortality of mice started. We conjectured that malignant ascites, metastasis in liver and tumor nodules on the omenta and mesentery were considered as the causes of death (Fig. 5). Our data demonstrated that oral administration of APW could not only inhibit tumor growth, but also liver and lung metastasis. Significantly, APW treatment enhanced the inhibitory effects of cisplatin plus 5-FU on tumor growth (Fig. 6). Although this enhanced inhibitory effect had not been observed in liver metastasis, the levels of ALT and LDH in the group treated by combination of APW, cisplatin and 5-FU were the lowest, implying that APW in combination with chemotherapeutics improved the liver function (Fig. 7B and C). Previous study reported that *A. paniculata* had benefits against liver damage caused by agents with different hepatotoxic mechanisms^13, 30^. Therefore, by demonstrating reduction of ALT and LDH, we have proven that APW improved the liver burden from metastasis and chemotherapeutics. From the data of CK levels, APW alone did not induce significant heart damage, but cisplatin plus 5-FU did. Moreover, APW combined with these chemotherapeutics attenuated their toxicities on the heart. This is the first study to illustrate some side effects from chemotherapeutics would be attenuated by APW combination (Fig. 7). In fact, there are many cancer patients asking for the administration of Chinese herbal medicines during chemotherapy, evidences from pre-clinical studies are urgently needed to prove the use of Chinese herbal medicines may not cause additional side effects.

The simultaneous reduction in the numbers of red and white blood cells, as well as platelets was known as a medical condition called pancytopenia, which was usually caused by chemotherapeutics for malignancies. On the other hand, anemia is a common side effect in cancer patients with chemotherapy^31^. Therefore, the counts of red blood cells, white blood cells and platelets as well as mean corpuscular hemoglobin concentration (MCHC) and hemoglobin concentration of the mice were evaluated after APW and/or chemotherapeutics treatments. Results showed that cisplatin plus 5-FU treatments cause pancytopenia and anemia. Although the combined APW with cisplatin plus 5-FU treatment could not improve the condition of pancytopenia and anemia, APW did not induce any hematological side effects in this tumor-bearing model (Fig. 7E–I). From the results of present study, we demonstrated the safety and side effects of Cisplatin + 5-FU combined with APW in pre-clinical model. We hope this information and further clinical studies can provide scientific evidences for patients and healthcare practitioners on the adjuvant values of Chinese herbal medicines in cancer management.

The adjuvant values of AP in metastatic EC treatment has been fully elucidated in the present study, which included 1) the intestinal absorption characteristics of APW; 2) the proven efficacy of absorbed components of AP in esophageal cancer cells; 3) the beneficial effects of AP combined with chemotherapeutics; 4) the ameliorated side effects by combined treatment and 5) the identification of some molecular mechanisms involved in AP’s effects.

In conclusion, some diterpenes and flavonoids exist in APW were transported through the Caco-2 monolayer, implying that they could be absorbed by the human gastrointestinal system. This is the first report of the absorbed components from APW that exerted inhibitory activities on the invasion and migration of esophageal cancer cells. The suppression of TM4SF3, HER2, CXCR4, MMP2 and the NFκB-mediated regulation of MMP9 expression were involved as the underlying mechanisms of its inhibitory effects. Oral administration of APW inhibited tumor growth and metastasis to liver and lungs in intraperitoneal xenograft-bearing mice without obvious toxicity. These effects may be responsible for the prolongation of survival induced by APW. Moreover, APW treatment enhanced the inhibitory effects of cisplatin plus 5-FU on tumor growth, and improved the side-effects induced by these chemotherapeutics. This is the first study to illustrate the safety and advantages of the combined use of the herbal medicine AP with chemotherapeutics in pre-clinical setting. Based on our results, APW should be considered as a promising candidate for further clinical trial to develop into an efficacious adjuvant treatment option for EC.

## Materials and Methods

### Materials

The raw herb *A. paniculata*, originated from Mainland China was purchased from a supplier in Hong Kong. Authentication of the raw herb was performed as described previously^16^. Authenticated herbal voucher specimen (number: 3435) was deposited in the museum of the Institute of Chinese Medicine, The Chinese University of Hong Kong. The powder of AP aqueous extract (APW) was prepared according to previous method^16^ and stored in desiccators at 4 °C. APW was dissolved in culture medium and subsequently filtered with 0.2 μm filter for *in vitro* studies. For *in vivo* studies, it was dissolved in distilled water. Chemotherapeutics cisplatin and 5-FU were purchased from Wako Pure Chemical Industries (Tokyo, Japan) and dissolved in saline before administration.

Human colonic adenocarcinoma cells Caco-2 and esophageal squamous carcinoma cells EC-109 were purchased from American Type Culture Collection (Manassas, VA, USA) and Cell Bank of Type Culture Collection of Chinese Academy of Sciences (Beijing, China), respectively. All cell culture reagents were purchased from Life Technology, USA. 3-(4,5-dimethylthiazol-2yl)2,5-diphenyltetrazolium bromide (MTT) were obtained from Sigma-Aldrich, USA. NFκB p65 (pS536) and NFκB p65 (Total) *in vitro* SimpleStepELISA kit, the primary antibodies against TM4SF3, MMP9 and CXCR4 were purchased from Abcam (Cambridge, UK). Primary antibodies against p-NFκB p65 (pS536), NFκB, HER2 and MMP2 were obtained from Cell Signaling Technology, USA.

### Cell line culture

Caco-2 cells were maintained in Dulbecco’s modified Eagle’s medium containing 10% (v/v) heat-inactivated fetal bovine serum (FBS), 1% non-essential amino acids, 100 U/ml penicillin and 10 U/ml streptomycin. EC-109 cells were cultured in RPMI 1640 medium with 10% (v/v) heat-inactivated FBS, 100 U/ml penicillin and 10 U/ml streptomycin. The cells were incubated at 37 °C in a humidified atmosphere with 5% CO_2_. When the cells reached 80% confluence in a culture flask, trypsin-EDTA (0.25%) was used to detach the cells and they were used in various experiments.

### Caco-2 monolayer transport assay

Caco-2 cells were seeded at a density of 3 × 10^5^ cells/well in six-well plates with Transwell^®^ inserts (0.4 μm pore size; Transwell Costar, Corning, USA) and cultured for 21 days prior to transport experiments. The integrity of the monolayer was monitored by measuring the transepithelial electrical resistance (TEER) with an epithelial voltohmmeter (World Precision Instruments, Inc., USA) both before and after the transport experiments. To carry out the transport experiment, Transwell^®^ inserts were firstly washed twice and then equilibrated with warmed HBSS transport buffer (pH 6.8) at 37 °C in a 5% CO_2_, 95% humidified atmosphere for 15 min. Then, 400, 800, 1600, 3200 or 6400 μg/ml APW were added on the apical side of the monolayer and incubated at 37 °C for 2 h. The above concentrations were chosen based on preliminary cytotoxicity test of APW on Caco-2 cells using MTT assay (data not shown). At the end of the experiment, HBSS buffer in the basolateral compartment, which contains the AAPC, was collected, lyophilized and reconstituted in methanol for HPLC or LC-MS analysis according to previous methods^16^ for detection and quantification of diterpenes and flavonoids. For *in vitro* bioassays, the reconstituted basolateral buffer was blown dried by nitrogen termovap sample concentrator and then redissolved in culture medium.

### Cytotoxicity assay of AAPC on EC-109 cells

The esophageal cancer cells EC-109 (5 × 10^3^ cells/well) were seeded in 96-well flat-bottomed culture plates and incubated overnight. Then, 100 μl culture media, containing AAPC (which were originally transported from 400–6400 μg/ml APW), were added into the wells. The plates were incubated at 37 °C for another 48 h. Subsequently, the cell viability was assessed by colorimetric MTT assay as described previously^32^.

### Cell migration assay

The motility of EC-109 cells was assessed by the scratch wound assay^33^. EC-109 cells (2.5 × 10^5^/well) were seeded in 24-well plates and incubated overnight. The adherent cells were starved with 0.5% v/v FBS medium for 24 h. The monolayers of cells were scraped with a cross in the middle of each well with 200 μl pipette tips. After scraping the cells, the medium was changed to fresh 10% v/v FBS medium with AAPC (from 800, 1600 and 3200 μg/ml APW). To evaluate the effects of AAPC on SDF1α (stromal cell-derived factor 1α)−induced migration, another set of experiment was also carried out by adding 10% v/v FBS medium with AAPC plus 500 ng/ml of recombinant human SDF1α (R&D Systems, Minneapolis, USA) after scraping. Subsequently, the cells were incubated for 24 h and each well was photographed at 40x magnification under a light microscope (Olympus IX-71, Japan). The percentages of open wound area were measured and calculated using the TScratch software. Inhibition of motility was determined by the decrease in closed wound area as compared with control.

The cell migration ability of EC-109 was evaluated by the Boyden chamber assay with minor modification^33^. Briefly, EC-109 cell suspension (5 × 10^4^ cells in 100 μl medium) was added into each transwell filter chamber with 8 μm pore size (Corning, USA). At the same time, AAPC (from 800, 1600 and 3200 μg/ml APW) were dissolved in 100 μl media (with 0.5% v/v FBS) and added to the upper chambers. To assess the inhibitory effects of AAPC on the migration stimulated by SDF1α, another set of experiment was simultaneously carried out by adding 0.5% v/v FBS medium with AAPC plus 500 ng/ml of SDF1α to the upper chambers. Five hundred microliters of complete culture medium (with 10% v/v FBS), serving as chemoattractant medium, were added to the lower chamber. After incubation for 24 h at 37 °C, the cells would have migrated to the lower chamber. Cells on the bottom surface of the filter membrane (migrated) were fixed with methanol and stained with hematoxylin. Stained filters were photographed at 100x magnification under a light microscope (Olympus IX-71, Japan). The migrated cells were quantified by manual counting. The changes in number of migrated cells were expressed as a percentage of control values.

### NFκB activity assay

NFκB activity was determined by NFκB p65 (pS536) + total NFκB p65 SimpleStep ELISA kit (Abcam, Cambridge, UK) according to the manufacturer’s instruction. Briefly, EC-109 cells (3 × 10^4^ cells/well) were seeded in 24-well plates overnight for attachment, and then incubated with AAPC (from 800, 1600 and 3200 μg/ml APW) for 24 h at 37 °C. Subsequently, the cells were washed with PBS twice and lysed for ELISA. Meanwhile, protein concentration was determined using BCA assay (Bicinchoninic acid kit, Sigma-Aldrich, USA). The NFκB and p-NFκB expression were normalised with the protein concentrations.

### Western blotting

EC-109 cells (3 × 10^5^ cells/well) were seeded in 6-well culture plates and incubated for 24 h to allow attachment. AAPC (from 800, 1600 and 3200 μg/ml APW) were then added into the wells and then incubated for another 24 h. Cells were washed with cold PBS twice and then lysed with radioimmunoprecipitation assay buffer [0.25% sodium deoxycholate, 1% Triton X-100, 0.1% SDS, 150 mM NaCl, 5 mM EDTA and 50 mM Tris–HCl (pH 7.5)] containing protease inhibitor cocktail (Roche Molecular Biochemicals, Switzerland). Subsequently, protein concentration was determined by BCA assay. Twenty-five microgram protein aliquots were placed in each lane and electrophoresed on 10% SDS-PAGE gels for 2 h at 100 V. Separated proteins were transferred electrophoretically to 0.45 μm polyvinylidene fluoride (PVDF) membrane (Immobilon, Millipore, USA) for 1.5 h at 90 V. The membrane was blocked with 5% (w/v) non-fat dry milk in tris buffered saline (TBS) for 1 h and subsequently incubated overnight at 4 °C with primary rabbit antibodies anti-HER2, MMP2, MMP9, CXCR4, TM4SF3, p-NFκB and NFκB at 1:1000 concentration, then washed with TBST (0.1% v/v Tween20 in TBS) and probed for 1 h with a horseradish peroxidase conjugated goat anti-rabbit IgG (1:2000; Life Technology, USA). To ensure equal protein loading, the membranes were subsequently stripped and incubated with monoclonal antibody against β-actin (1:10,000), and followed by a horseradish peroxidase conjugated goat anti-mouse IgG (1:5000). Protein bands were visualized by reaction with enhanced chemiluminescence assay kit (GE Healthcare, USA).

### Real time-PCR analysis

EC-109 cells (3 × 10^5^ cells/well) were seeded in 6-well culture plates and incubated for 24 h to allow attachment. AAPC (from 800, 1600 and 3200 μg/ml APW) were then added into the wells and incubated for another 24 h. After treatment, cells were extracted using TRIzol Reagent (Life Technology, USA) according to the manufacturer’s protocols. The RNA concentration was spectrophotometrically determined by a BioPhotometer (Eppendorf, NY, USA). To quantify the amount of mRNA of *TM4SF3*, *MMP9*, *HER2* and *CXCR4*, real time-PCR was carried out as previously described^16^. The specific gene mRNA levels were normalized to *GAPDH* and expressed as a fold change compared to the control group. All experiments were performed independently for three or four times. The sequences of the primers used are listed in Supporting Information Table S1.

### Intraperitoneal esophageal tumor xenograft-bearing mice model

Due to the significant anti-metastatic effect of AAPC *in vitro* (as shown in result section), further animal studies on APW were conducted using the intraperitoneal esophageal tumor xenograft-bearing mice. Male BALB/c nude mice (6–8 weeks of age) were provided by Laboratory Animal Services Center of The Chinese University of Hong Kong and were bred and maintained in pathogen-free conditions. The experiments were approved by the Animal Experimentation Ethics Committee of The Chinese University of Hong Kong (Ref. No. 12–078-MIS). All experimental methods in mice were carried out in accordance with the approved guidelines specified by the Animal Experimentation Ethics Committee of the Chinese University of Hong Kong. Intraperitoneal esophageal tumor xenograft-bearing mice model was applied to test the *in vivo* efficiency of APW alone or in combination with standard chemotherapeutics cisplatin and 5-FU.

#### Survival experiments

The survival of intraperitoneal esophageal tumor xenograft-bearing mice model was determined. Briefly, 5 × 10^6^ EC-109 cells in 200 μl PBS were inoculated intraperitoneally into the mice on day 0. On day 1, animals were randomized into 4 groups: 1) control (n = 9), 2) 1600 mg/kg APW alone (APW, n = 10), 3) combination of 3.0 mg/kg cisplatin and 85.0 mg/kg 5-FU (Cisplatin + 5-FU, n = 10), and 4) combination of 1600 mg/kg APW, 3.0 mg/kg cisplatin and 85.0 mg/kg 5-FU (APW + Cisplatin + 5-FU, n = 10). APW was oral-administered daily starting from day 1 for 60 days. On days 13 and 19, cisplatin and 5-FU were injected intraperitoneally into the mice of respective groups^34^. The survival rate of the mice was recorded during the 60 days period.

#### Anti-tumor and anti-metastatic efficacies of combined treatments

Intraperitoneal esophageal tumor xenograft-bearing mice were randomized on day 1 into the same 4 groups: control (n = 13), 1600 mg/kg APW alone (APW, n = 12), the combination of 3.0 mg/kg cisplatin and 85.0 mg/kg 5-FU (Cisplatin + 5-FU, n = 10), and the combination of 1600 mg/kg APW, 3.0 mg/kg cisplatin and 85.0 mg/kg 5-FU (APW + Cisplatin + 5-FU, n = 10). APW was orally administered daily for 21 days, and cisplatin and 5-FU were injected intraperitoneally on days 13 and 19. On day 22, the mice were anesthetized and whole blood was obtained by cardiac puncture. The animals were sacrificed by cervical dislocation and the tumor nodules in peritoneal cavity were collected, counted and weighed^35^. The sum of all tumors from intraperitoneal cavity of individual was tumor weight. Nodule with diameter greater than 2 mm was counted into the number of tumor nodules^35^. Plasma was collected by centrifugation of the blood (1700× g, 10 min, 4 °C) and stored at −80 °C. Concentrations of alanine aminotransferase (ALT), aspartate aminotransferase (AST) and lactate dehydrogenase (LDH) for assessment of liver damage, and creatine kinase (CK) for assessment of heart damage, were analyzed using respective enzyme assay kits according to the manufacturer’s instructions (Stanbio Laboratory, USA). In addition, the enzyme levels of a naive group (n = 12), in which no cancer cells were inoculated, were used for comparison to tumor-bearing groups. In another set of experiment with the same treatments, the counts of the red blood cells, white blood cells, platelets, hemoglobin levels and mean corpuscular hemoglobin concentration (MCHC) from mice were also measured on day 22.

#### Histology

Livers and lungs of the mice were dissected out after cervical dislocation and fixed in 10% buffered formalin. The samples were then processed for paraffin embedding and sectioned longitudinally at 5 μm thickness using a biocut rotary microtome machine (Shandon Finesse 325, Thermo Scientific, USA). The tumor burden in lung or liver was calculated from three levels of each paraffin-embedded tissue blocks. Each level of section was kept an interval of 100 μm longitudinally and collected onto gelatin-coated slides, then stained with haematoxylin & eosin. Stained sections were examined and photographed under a light microscope (Olympus IX71, Japan). Evaluation of liver or lung metastasis was carried out according to previous studies^36, 37^. In brief, the tumor area and the organ area in each slide were measured by the ImageJ software (NIH, USA). The tumor burden in liver, defined as the tumor area, was calculated from the section and expressed as an average tumor area per group in absolute units (mm^2^). The tumor burden in lung was defined as the average percentage of tumor area to lung area.

### Statistical analysis

Data were expressed as mean ± standard deviation (S.D.) for *in vitro* studies, and as mean ± standard error of the mean (S.E.M.) for *in vivo* studies. One way analysis of variance (ANOVA) followed by *post-hoc* Dunnett’s test were used to compare the treatment groups and the control group. One-way ANOVA followed by *post-hoc* Tukey’s multiple comparison tests were used to determine significant differences among all groups. The Student’s t-test was performed to compare the difference between two groups. For the survival data, they were analyzed using Prism 5 and differences among treatment groups were compared using log-rank (Mantel–Cox) test. Statistical analyses were conducted using a GraphPad Prism5.0 software package (GraphPad Software Inc., San Diego, CA). Differences were considered to be statistically significant when *p* < 0.05.

## Electronic supplementary material

Supporting Information
