# Supplementary material for: The adjuvant value of Andrographis paniculata in metastatic esophageal cancer treatment – from preclinical perspectives
Source: Sci Rep. 2017 Apr 12;7:854. doi: 10.1038/s41598-017-00934-x (PMC5429803; doi:10.1038/s41598-017-00934-x)
Supplement: Supplementary file 1 — Supporting Information [file 41598_2017_934_MOESM1_ESM.pdf]

The adjuvant value of *Andrographis paniculata* in metastatic esophageal cancer treatment – from preclinical perspectives

Lin Li[1], Grace Gar-Lee Yue[2], Julia Kin-Ming Lee[2], Eric Chun-Wai Wong[2], Kwok-Pui Fung[2,3], Jun Yu[4], Clara Bik-San Lau[2,*], Philip Wai-Yan Chiu[1,*]

[1] Department of Surgery; [2] Institute of Chinese Medicine and State Key Laboratory of Phytochemistry and Plant Resources in West China (CUHK); [3] School of Biomedical Sciences; [4] Department of Medicine and Therapeutics and State Key Laboratory of Digestive Disease, The Chinese University of Hong Kong, Shatin, New Territories, Hong Kong.

Supporting Information

Table S1. Sequence of specific PCR primers of different target genes

Genes	Forward Primer	Reverse Primer
TM4SF3	ATAGATATCGACAAGCCTGTAACGAA	GATCTCGAGGTTCCCGATCTGG
MMP9	Hs_MMP9_1_SD QuantiTect Primer Assay (QIAGEN)	
HER2	GAGCCGCGAGCACCCAAGT	TCCATTGTCTAGCACGGCCA
CXCR4	GGCAGAGGAGTTAGCCAAGAT	CTCCTCCCCATCTTTTCCCAT
GAPDH	CGAGATCCCTCCAAAATCAA	GGTGCTAAGCAGTTGGTGGT
